# Presentation management and outcome of phlegmonous and inflammed appendicitis in children in Ethiopia: retrospective review

**DOI:** 10.1186/s12893-023-02191-4

**Published:** 2023-09-14

**Authors:** Belachew Dejene Wondemagegnehu

**Affiliations:** College of Health Sciences Department of Surgery, Addis Ababa University, Addis Ababa, Ethiopia

**Keywords:** Pediatric Appendicitis, Uncomplicated Appendicitis, Phlegmonous, Inflamed

## Abstract

**Background:**

Acute appendicitis is the leading cause of emergency pediatric surgical admissions in the world. The diagnosis is may be difficult and is often dependent on clinical parameters. This study was aimed at reviewing the clinical presentations, the management and outcomes in children with inflamed and phlegmonous appendicitis with reference to the operative findings.

**Methods:**

The records of 211 children 5 to 15 years of age who were operated for acute appendicitis with intra operative findings of inflamed or phlegmonous appendicitis who met inclusion criteria were entered and analyzed using SPSS (IBM) V.26. Descriptive and regression tests were done with *p* < 0.05 considered statistically significant.

**Results:**

Of 211 children with inflamed and phlegmonous appendicitis, the M: F was 1.48:1 with a median age of 11 years. 58.3% of them presented within 24 h with the commonest symptoms being right lower abdominal pain, anorexia, and vomiting (96.2%,96.2%, 85.3%,) respectively. 96.7% of them had right lower abdominal tenderness. 73% had neutrophils ≥ 75%, and of 171 patients who had abdominal ultrasound scan, 97.7% showed appendiceal diameter ≥ 6 mm. Intraoperatively 56.4% of them were found to have phlegmonous appendicitis. In a retrospective Pediatric Appendiceal Score, only 52.6% of patients fall into the high-risk category, who could be confirmed on preoperative clinical assessment. Postoperatively 90% of them discharged improved with a mean hospital stay of 2.26(SD = 0.9) days. There was no association between the sex of the child and the intraoperative finding of inflamed or phlegmonous appendicitis (*p* = 0.77).

**Conclusion:**

Pediatric appendicitis affects more male children in their second decade of life. Most had phlegmonous appendicitis and presented within 24 h. Duration of illness has little effect on the progress of appendicitis. Surgical management is safe for inflamed and phlegmonous appendicitis with a reasonable hospital stay and a low rate of complications.

## Introduction

Acute appendicitis is the leading cause of surgical emergency admissions in the world [1, 2] and the lifetime risk of developing appendicitis is estimated to be 8.7% for boys and 6.7% for girls with a peak occurrence in the second or third decade of life affecting more males [3]. It is responsible for significant complications, particularly in children of the developing world. The patients often present with non-specific symptoms and signs mimicking other pathologies that may make the diagnosis difficult, with a negative appendectomy rate of 8.4% [4].

A correct and timely diagnosis of acute appendicitis is very important in reducing the complications and negative appendectomy rates, for this reason several diagnostic scores have been introduced in the last few decades but none has gained wide acceptance because of variability of the outcomes [5]. The diagnosis of acute appendicitis is the total of history, and physical findings supplemented with laboratory investigations and selectively focused imaging. Some studies indicated that a surgeon’s experience is a better predictor of uncomplicated appendicitis than clinical or imaging findings [6]. The role of Ultrasound (US) and other imaging modalities remains a point of debate [7].

Of the scoring systems proposed to evaluate appendicitis in children presenting with acute abdominal pain, Pediatric Appendicitis Score (PAS) and Alvarado scores are relatively regarded as better tools to use as a guide to assist in the decision of the management of a child with abdominal pain [7, 8].

Even if recent evidence recommends medical management as an alternative treatment of patients who have clinical and imaging diagnosis of localized appendicitis, appendectomy remains the mainstay of treatment for uncomplicated appendicitis to prevent further complications [9, 10].

There are variable study findings worldwide in the presentation, diagnosis, management and out come of appendicitis [11, 12]. The aim of this study is to retrospectively evaluate the efficacy of frequently assessed preoperative clinical parameters, investigations and commonly used appendicitis scores (Alvarado and PAS) in the diagnosis of phlegmonous and inflamed appendicitis with reference to intra-operative findings.

## Methods and materials

### Study design and setting

It is a retrospective cohort institution-based study conducted on all children (*N* = 416) 5 to 15 years of age who underwent appendectomy between January 1^st^,2018 to December 31^st^, 2020 at Tikur Anbesa Specialized Hospital and its affiliate Menelik-II Specialized Hospital, where fulltime surgical service is given by Pediatric surgery residents, Fellows, and Consultants.

### Data management

After Checklist was prepared and medical records examined, data were coded and entered into SPSS(IBM) V. 26 based on required baseline information on demography, pre-hospital duration of illness, admission date, all presenting symptoms, admission physical examination findings, preoperative laboratory and ultrasound results, intraoperative findings, pre and postoperative management and post-operative course including the overall hospital stay. The findings also summed up for PAS and Alvarado scores. The type of appendicitis was sorted based on the intra-operative description of the appendix with signs of inflamed appendix (*hyperemic and or swollen appendix without mass formation*) or phlegmon (*appendiceal inflammatory mass with out an apparently defined abscess, perforation, or gangrene*). Histologic finding was not included in the study as it was done only for few children with specific indications.

### Study population

**Fig. 1:**
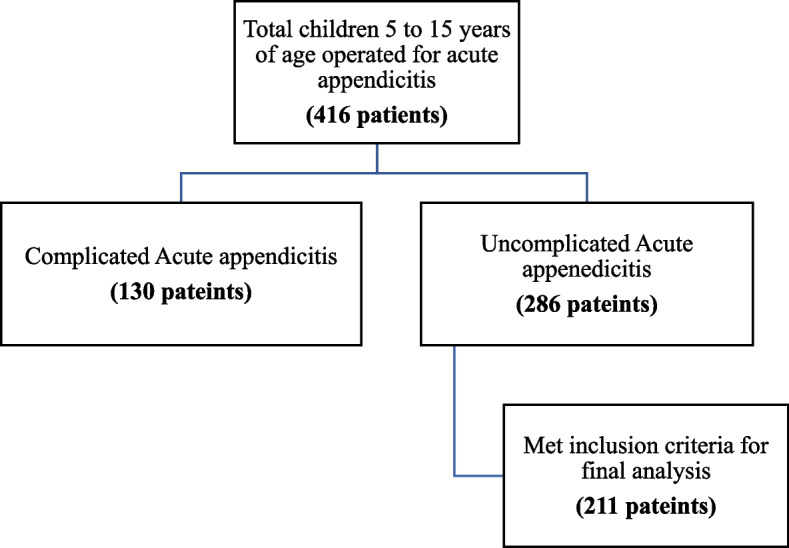
Flow diagram showing the process of selection of study population in a retrospective review of children five to fifteen years of age operated for acute inflamed and phlegmonous appendicitis at Tikur Anbessa and Minilik-II Tertiary Hospitals from January 1^st^, 2018 to December 31^st^, 2020, Addis Ababa, Ethiopia

Two hundred eighty-six children 5 to 15 years of age who had an intra-operative description of inflamed and phlegmonous appendicitis were considered and 211 patients with complete medical records who met the inclusion criteria were taken for analysis, as shown on the flow diagram (Fig. 1).

#### Inclusion criteria

Those children age 5 to 15 years with a duration of illness within 48 h and confirmed intraoperative findings suggesting inflamed or phlegmonous appendix.

#### Exclusion criteria

Those children age less than 5 and greater than 15 years with a duration of illness more than 48 h and complicated cases as noted from intra-operative findings. Patients with incomplete medical records and those who took pre-hospital visit medications were also excluded from the study.

### Data analysis

Data was analyzed using SPSS (IBM) V.26 Windows software. Descriptive analysis was performed on demographic variables and logistic regression to assess the odds ratio based on characteristics of the variables, and the confidence interval of statistical associations to determine the association between dependent and independent variables, Value of *p* < 0.05 was considered statistically significant and the results were presented using text, tables, and a figure.

## Results

A total of 416 children, between the ages of 5 to 15 years, were operated on for all types of acute appendicitis. Those with inflamed and phlegmonous appendicitis account for 286(68.8%) of whom 211 met the inclusion criteria for analysis. Of the 211 cases selected 126(59.7%) were male with M: F of 1.48:1, and median age of 11 years. The mean duration of illness was 29.4 h with the common presenting symptoms reported of right lower abdominal pain in 203(96.2%) with an associated shift of abdominal pain in 73% of cases and vomiting in 85.3% of cases. A significant number of them (96.2%) presented with loss of appetite and 52.6% of them reported low-grade fever (Table 1).

**Table 1:** Demographic and presenting symptoms of inflamed and phlegmonous appendicitis in children aged 5 to 15 years

**Variables**	**Intraoperative findings**			
	**Categories**	**Inflamed appendix (*n* = 92)**	**Phlegmonous appendix (*n* = 119)**	**Totals**
		***n* (%)**	***n* (%)**	***n* (%)**
Age	*5 to 10*	41(19.4)	59(28.0)	100 (47.4)
	> *10*	51(24.2)	60(28.4)	111 (52.6)
	***Total***	**92(43.6)**	**119(56.4)**	**211(100)**
Sex of the child	*Male*	56(26.5)	70(33.2)	126 (59.7)
	*female*	36(17.1)	49(23.2)	85 (40.3)
	***Total***	**92(43.6)**	**119**	**211 (100)**
Duration of symptoms	≤ *24 h*	65(30.8)	58(27.5)	123 (58.3)
	> *24 h*	27(12.8)	61(28.9)	88 (41.7)
Rt. LQ abdominal pain	*Yes*	90(42.7)	113(53.5)	203 (96.2)
	*No*	2(0.9)	6(2.8)	8 (3.7)
Shift of abdominal pain	*Yes*	60(28.4)	94(44.5)	154 (73.0)
	*No*	32(15.2)	25(11.8)	57 (27.0)
Episodes of vomiting	*Yes*	67(31.8)	113(53.5)	180 (85.3)
	*No*	25(11.8)	6(2.8)	31 (14.7)
Loss of appetite	*Yes*	86(40.8)	117(55.4)	203 (96.2)
	*No*	6(2.8)	2(0.9)	8 (3.7)
Febrile episodes	*Low grade*	45(21.3)	66(31.2)	111 (52.6)
	*High grade*	4(1.9)	6(2.8)	10 (4.7)
	*No*	43(20.4)	47(22.3)	90 (42.7)

Frequency distribution of demographic and presenting symptoms of 211 children operated on for acute inflamed and phlegmonous appendicitis at Tikur Anbessa and Menellek II tertiary hospitals from Jan. 2018 to Dec.2020

During physical examination only 37.9% of them exhibited pulse rate above 100/minute, most of them (77.3%) showed temperature records of less than 37.2 ^o^ C, and a significant number of cases (96.7%) were found to have some degree of right lower quadrant tenderness (Table 2).

**Table 2:** Frequency of clinical signs of inflamed and phlegmonous acute appendicitis

Variables	Categories	Intraoperative findings		Total
		**Inflamed appendicitis (*n* = 92)**	**Phlegmonous Appendicitis (*n* = 119)**	
		***n* (%)**	***n* (%)**	
Pulse rate at admission	< *80*	2(0.9)	2(0.9)	4(1.8)
	*80 to 100*	37(17.5)	39(18.5)	76(36.0)
	*101 to 120*	39(18.5)	53(25.1)	92(43.6)
	> *120*	14(6.6)	25(11.8)	39(18.4)
Temperature at admission	< *37.2*	77(36.5)	86(40.8)	163(77.3)
	*37.3 to 38*	10(4.7)	24(11.4)	34(16.1)
	*.* > *38*	5(2.4)	9(4.3)	14(6.7)
Abdominal tenderness	*Yes*	86(40.8%)	118(55.9)	204(96.7)
	*No*	6(2.8%)	1(0.4)	7(3.3)

Frequency distribution of clinical signs at the presentation of 211 children operated for acute inflamed and phlegmonous appendicitis at Tikur Anbessa and Menellek II tertiary hospitals from Jan. 2018 to Dec.2020

Seventy-one percent of them were recorded to have a WBC count of more than 10,000/mm^3^ with a mean count of 12,918/mm^3^ and 154(73%) had Neutrophils ≥ 75% with a mean of 78.6%. Of 171 patients whose Ultrasound records were examined 167(97.7%) had appendiceal size greater than 6 mm and a mean of 8.3 mm.

Open appendectomy was done for all cases with finding of 56.4% phlegmonous and 43.6% inflamed appendicitis. The appendix was located in 104(49.3%) retro cecal with 67(31.7%) of them sub-Cecal and the rest in the pelvic, pre-ileal, and sub-hepatic positions.

Postoperatively 205(97.1%) of them took antibiotics during their hospital stay and only 33.6% of them were given oral antibiotics upon discharge and the average hospital stay was 2.6 days (SD 0. 917). The postoperative course for most of them (90%) was uneventful and most of them were followed up for one month, with only 21(10%) developing minor wound complications and other problems related to neither the disease process nor the management of appendicitis.

Of the 211 patients' records computed 52.2% fall in high-risk category for PAS and 40.3% for Alvarado with a mean score of 7.4(SD = 1.920) and 7.66(SD = 1.92) respectively (Table 3).

**Table 3:** Retrospectively calculated PAS and Alvarado scores of cute inflamed and phlegmonous appendicitis

**Score**	**Points**	**Intra-operative findings**			
		**Risk category**	**Inflamed (*n* = 92)** ***n* (%)**	**Phlegmonous (*n* = 119)** ***n* (%)**	**Total**
**Retrospectively calculated PAS Score**	*8 to 10*	High risk	38(18.0)	73(34.6)	111(52.6)
	*4 to 7*	Intermediate	46(21.8)	46(21.8)	92(43.6)
	*1 to 3*	Low risk	8(3.8)	0	8(3.8)
**Retrospectively calculated Alvarado score**	*9 to 10*	Very likely	25(11.8)	60(28.4)	85(40.3)
	*7 to 8*	Probable	35(16.6)	42(19.9)	77(36.5)
	*5 to 6*	Possible	16(7.6)	15(7.1)	31(14.7)
	< *4*	unlikely	16(7.6)	2(0.9)	18(8.5)

Retrospectively calculated Pediatric Appendiceal Score and Alvarado Score to predict acute uncomplicated appendicitis in 211 children operated for acute inflamed and phlegmonous appendicitis at Tikur Anbessa and Menellek II tertiary hospitals from Jan. 2018 to Dec.2020

Logistic regression was run to look for clinical parameters in predicting the inflamed or phlegmonous appendicitis variables entered showed good model fit (*p* = 0.354, chi-square = 3.255, df = 3). The sensitivity of the model in predicting intraoperative findings inflamed or phlegmonous appendicitis was high indicating most of them could be predicted by preoperative assessment. The likelihood of making inflamed or phlegmonous appendicitis within 48 h of illness was high for right lower quadrant abdominal tenderness, Anorexia, vomiting, the shift of pain, admission temperature record of > 37.2 ^0^C, and sex of the patient (Table 4).

**Table 4:** Likelihood of making the diagnosis of acute inflamed or phlegmonous appendicitis within 48 h of illness

Clinical Variable	AOR	CI
Sex	1.003	95% CI of 0.475 to 2.135
Anorexia	11.504	95% CI of 1.039 to 127.430
Vomiting	6.740	95% CI of 1.690 to 26.879
Shift of abdominal pain	3.178	95% CI of 1.257 to 8.036
Right lower abdominal tenderness	11.536	95% CI of 1.039 to 128.141
Temperature > 37.2^0^C	1.540	95% CI of 0.302 to 7.858

Likelihood of making the diagnosis of Pediatric Uncomplicated Acute Appendicitis within 48 h of illness in children 5 to 15 years of age in 211 children operated for acute inflamed and phlegmonous appendicitis at Tikur Anbessa and Menellek II tertiary hospitals from Jan. 2018 to Dec. 2020

Prediction of the inflamed appendix is significantly noticed in those who presented with a duration of less than 24 h, vomiting, and Neutrophil < 75% with *p* < 0.001 for all, and correlation analysis indicated no association between sex and type of appendicitis (*p* = 0.765).

## Discussions

Even though several studies have been conducted regarding the general knowledge of acute appendicitis, only few studies have been done relating intraoperative findings with clinical parameters and the contribution of individual parameters in the prediction and diagnosis of inflamed and phlegmonous appendicitis.

The study found that there is slight male predominance mostly affecting children around eleven years and most of them presented with right lower abdominal pain, the shift of pain, vomiting, loss of appetite, and low-grade fever. Variable degree of right lower quadrant abdominal tenderness was the predominant finding and a significant number of patients demonstrated a WBC count above 10,000/mm^3^, neutrophil percentage of ≥ 75%, and Ultrasound appendiceal diameter of ≥ 6 mm, indicating a higher predictive ability of these parameters in the diagnosis of inflamed or phlegmonous appendicitis, whereas the changes in the vital signs were unremarkable showing little contribution in the prediction of the likelihood of the two forms of acute appendicitis. Though most presented within 24 h, in contrast to the general belief that phlegmon is a late process of early appendicitis, there was a higher number of phlegmonous appendicitis implying delay in diagnosis of more than 24 h might carry a potential risk of complications. Regarding appendiceal scoring systems, relatively a higher number of retrospectively assessed PAS group were in the high-risk category, making it the better scoring system as compared to Alvarado in the diagnosis of children with inflamed or phlegmonous appendicitis. The Post-operative course in the study cases was uneventful for most and discharged improved with a reasonable hospital stay, denoting open surgical management is still a viable mode of intervention in the developing world where there is no luxury of laparoscopic and medical management.

Of the 211 pediatric patients operated for inflamed and phlegmonous appendicitis 59.7% were male with a M:F ratio of 1.48 similar to other studies [13, 14] and the most affected age was around eleven years which was almost in line with other reports of 10.5 years, and 10yrs [15, 16] In this study, no seasonal variation was noted against 11.3% increased occurrence during the summer season reported in a similar studies [2, 3, 17].

The mean duration of illness was 29.4 h, comparable with other studies [18, 19]. 96.2% of them presented with right lower abdominal pain with 73% of them reporting a shift of pain and 85.3% with vomiting consistent with others. 96.2% of cases in this study reported anorexia which had significant variability among different studies. The result is comparable with Huang TH and colleagues [19] who found higher frequencies of anorexia, nausea, vomiting, and leukocytosis in children but was quite far from reports of only 28.2% by Seah and Ng [20], and a lower likely hood ratio reported by another study. Most of the findings were consistent with other studies except the anorexia in which different studies demonstrated varying results. Considering the finding in this study, the Author feels that enough emphasis has not been given like other symptoms in most literature to the contribution of anorexia in the prediction and diagnosis of inflamed and phlegmonous appendicitis. During the physical examination, only 37.9% of them exhibit pulse rate above 100/minute, and a significant number of them (77.3%) showed temperature records of less than 37.2 o C indicating that the presence or absence of fever has no effect on the probability of occurrence of appendicitis [21], and 96.7% had various degrees of right lower quadrant tenderness indicating the reason why it was given higher weight by most Appendicitis predictive scores [5, 8, 22, 23].

According to Samuel M., the Mean white blood cell count was 13.5 × 10^3^ (SD = 4000) in inflamed appendicitis [8], and in another study, more than 80% showed a rise in WBC count and high neutrophil percentage with a left shift in 72% of cases [20] which is comparable with this study showing mean WBC count of 12,918 (SD = 4,950) and Neutrophil mean percentage of 78.6%. The slightly higher percentage in this study is because of relatively more numbers of phlegmonous appendicitis.

In a retrospectively assessed PAS, only 52.2% were at high risk with a mean score of 7.4 (SD = 1.920) which is higher than other studies with a PAS mean of 5.8 (SD = 1.4) [24], and in Alvarado, only 40.3% had a high-risk result with a mean score of 7.66(SD = 1.92), in another study only PAS ≥ 8 was making the prediction of high-risk patients for Acute Appendicitis [21].

Literature is lacking on the intraoperative patterns of inflamed vs phlegmonous appendicitis. In this study, there were 56.7% found to have phlegmonous appendicitis with the majority (58.3%) presented within 24 h which is against the common belief that phlegmon is a late event in the early stage of appendicitis. Regarding intra-operative positions of the appendix nearly half of them were retro-Cecal which were completely different from figures in the literature [25].

Postoperatively 97.1% of them took antibiotics during their hospital stay with a reasonable hospital stay of 2.6 days (SD = 0. 917). The postoperative course for the majority of them (90%) was uneventful with no recorded mortality and the rest with minor wound infections and complications unrelated to appendicitis, indicating surgical management is still a viable option for patients with acute inflamed and phlegmonous appendicitis.

## Conclusions

The variabilities in the findings among different assessment methods in the prediction of inflamed or phlegmonous appendicitis are the results of underemphasis of the relative contributions of clinical symptoms and signs. Right lower abdominal pain with any degree of tenderness, anorexia, vomiting and nausea predicted well the occurrence of acute inflamed and phlegmonous appendicitis and duration of illness may not be the major factor in the progression of appendicitis. Some clinical variables, like anorexia, were understated in many studies despite their occurrence in a significant proportion. Surgical management remains a viable option of management for Paediatric acute appendicitis with reasonable hospital stays and a low rate of complications.

### Limitations of the study

It is a retrospective study with missing data for some of the variables limiting its clinical applicability and needs to be conducted on a larger scale.

## Data Availability

Data are available upon reasonable request from the corresponding author.
